# Sex differences in subjective cognition among middle‐aged and older Hispanics/Latinos: Findings from the HCHS/SOL and SOL‐INCA

**DOI:** 10.1002/alz70860_107161

**Published:** 2025-12-23

**Authors:** Ariana M Stickel, Wassim Tarraf, Mariana Montoya, Sayaka Kuwayama, Carlos E.E. Araujo Menendez, Zvinka Z. Zlatar, Linda C Gallo, Krista M Perreira, Haibo Zhou, Martha L Daviglus, Amber Pirzada, Paola Filigrana, Bonnie Levin, Hector M González, Sarah J Banks

**Affiliations:** ^1^ San Diego State University, San Diego, CA, USA; ^2^ Wayne State University, Detroit, MI, USA; ^3^ University of California, San Diego, La Jolla, CA, USA; ^4^ SDSU/UC San Diego Joint Doctoral Program in Clinical Psychology, San Diego, CA, USA; ^5^ University of North Carolina, Chapel Hill, NC, USA; ^6^ University of North Carolina, Chapel Hill, Chapel Hill, NC, USA; ^7^ University of Illinois at Chicago, Chicago, IL, USA; ^8^ University of Illinois, Chicago, IL, USA; ^9^ Albert Einstein College of Medicine, Bronx, NY, USA; ^10^ University of Miami Miller School of Medicine, Miami, FL, USA

## Abstract

**Background:**

Subjective cognitive decline (SCD) is recognized as a possible prodrome to Alzheimer's disease and related dementias. Existing literature points to sex/gender differences in SCD, yet the extent to which these differences apply to underrepresented groups is unknown. Further, certain factors (e.g., cardiovascular disease [CVD] risk, mood disorders, acculturation) may change the relationships between sex and SCD. Therefore, we investigated the relationship between sex and SCD and its modifiers in a diverse group of Hispanic/Latino adults.

**Method:**

Participants included 5,985 Hispanic/Latino adults (3,857 females; ages 50+ years) from the Study of Latinos ‐ Investigation of Neurocognitive Aging (SOL‐INCA), an ancillary study of the Hispanic Community Health Study/Study of Latinos. Our exposure of interest was sex (male/female). SCD was operationalized as continuous overall and domain‐specific (executive functioning, memory, language, visuospatial) self‐reported cognitive change based on the Everyday Cognition 12‐item questionnaire. Modifiers included CVD risk, anxious‐depression symptoms, social acculturation, and language acculturation. Survey‐adjusted linear regressions tested the associations between sex and SCD in three sets of models: 1) crude; 2) age, education, Hispanic/Latino heritage, and field center adjusted; and 3) fully adjusted (i.e., adding main effects of modifier variables). Then, in the fully adjusted models, we included interactions terms to individually test each modifier.

**Results:**

Participants were 63‐years old on average and 39% reported less than high school education. Adjusting for demographic covariates, males reported less SCD globally (b=‐0.11, CI:‐0.19,‐0.03) and across all domains except executive functioning. In fully adjusted models, sex differences in language and visuospatial SCD domains remained significant. Tests of effect modification (significant interaction effects) and plotting marginal means indicated that sex differences were less pronounced for 1) language SCD at lower levels of CVD risk, and 2) visuospatial SCD with higher language acculturation (i.e., English language use in more contexts).

**Conclusion:**

Consistent with the existing literature, female Hispanic/Latino adults tended to self‐report more cognitive decline, particularly in language and visuospatial domains. However, these differences were minimized in the context of lower cardiovascular disease risk and higher language acculturation, respectively. Future research should investigate the degree to which biopsychosocial factors impact these nuanced relationships between sex and SCD.

